# Deciphering the Effect of Different Genetic Variants on Hippocampal Subfield Volumes in the General Population

**DOI:** 10.3390/ijms24021120

**Published:** 2023-01-06

**Authors:** Kevin Kirchner, Linda Garvert, Katharina Wittfeld, Sabine Ameling, Robin Bülow, Henriette Meyer zu Schwabedissen, Matthias Nauck, Henry Völzke, Hans J. Grabe, Sandra Van der Auwera

**Affiliations:** 1 Department of Psychiatry and Psychotherapy, University Medicine Greifswald, 17475 Greifswald, Germany; 2 German Centre for Neurodegenerative Diseases (DZNE), Site Rostock/Greifswald, 17475 Greifswald, Germany; 3 Interfaculty Institute for Genetics and Functional Genomics, University Medicine Greifswald, 17475 Greifswald, Germany; 4 German Centre for Cardiovascular Research (DZHK), Partner Site Greifswald, 17475 Greifswald, Germany; 5 Institute for Diagnostic Radiology and Neuroradiology, University Medicine Greifswald, 17475 Greifswald, Germany; 6 Department Pharmaceutical Sciences, University of Basel, 4056 Basel, Switzerland; 7 Institute of Clinical Chemistry and Laboratory Medicine, University Medicine Greifswald, 17475 Greifswald, Germany; 8 Institute for Community Medicine, University Medicine Greifswald, 17475 Greifswald, Germany

**Keywords:** hippocampal subfields, verbal memory, *APOE*, *BDNF*, *5-HTTLPR*, polygenic score, general population, trisynaptic circuit, candidate genes, depression

## Abstract

The aim of this study was to disentangle the effects of various genetic factors on hippocampal subfield volumes using three different approaches: a biologically driven candidate gene approach, a hypothesis-free GWAS approach, and a polygenic approach, where AD risk alleles are combined with a polygenic risk score (PRS). The impact of these genetic factors was investigated in a large dementia-free general population cohort from the Study of Health in Pomerania (SHIP, *n* = 1806). Analyses were performed using linear regression models adjusted for biological and environmental risk factors. Hippocampus subfield volume alterations were found for *APOE* ε4, *BDNF* Val, and *5-HTTLPR* L allele carriers. In addition, we were able to replicate GWAS findings, especially for rs17178139 (*MSRB3*), rs1861979 (*DPP4*), rs7873551 (*ASTN2*), and rs572246240 (*MAST4*). Interaction analyses between the significant SNPs as well as the PRS for AD revealed no significant results. Our results confirm that hippocampal volume reductions are influenced by genetic variation, and that different variants reveal different association patterns that can be linked to biological processes in neurodegeneration. Thus, this study underlines the importance of specific genetic analyses in the quest for acquiring deeper insights into the biology of hippocampal volume loss, memory impairment, depression, and neurodegenerative diseases.

## 1. Introduction

The hippocampus is a functional and cytoarchitectural heterogeneous subcortical brain structure within the limbic system [1], and plays an important role in the execution of various brain functions, including episodic memory, learning, and spatial navigation [2,3]. Regarding cytoarchitecture, the hippocampus comprises diverse subfields, which are divided into the cornu ammonis (CA1, CA2/CA3, CA4); presubiculum, subiculum, and parasubiculum; dentate gyrus (granule and molecular layer); hippocampal fissure, fimbria, and tail; as well as the hippocampus–amygdala transition area (HATA) [1,4,5]. It has been widely shown that a loss of hippocampal volume [6,7] and bilateral hippocampal connectivity [8] is associated with the development of mild cognitive impairment (MCI) and Alzheimer’s disease (AD). Thereby, preliminary results emphasized that the specific subfields are more sensitive in predicting AD than the whole hippocampus [9]. Furthermore, alterations in hippocampal volume have been found throughout various psychiatric conditions, including depression and schizophrenia [10].

In addition to functionality and cytoarchitecture differences, several studies also indicated differences in the genetic structure of hippocampal subfield volumes [1,11]. A first review on the genetic influence of single-nucleotide polymorphisms (SNPs) on hippocampal subfields is provided by Vilor-Tejedor and colleagues [1]. The authors explicitly differentiate between genetic variants identified in biologically driven candidate gene approaches and those from hypothesis-free genome-wide association studies (GWAS). On the candidate gene side, their review includes prominent memory-associated variants, such as apolipoprotein E ε4 (*APOE* ε4), brain-derived neurotrophic factor Val^66^Met polymorphism (*BDNF*, rs6265), serotonin-transporter-linked promoter region (*5-HTTLPR*), kidney and brain expressed protein (*KIBRA*, rs17070145), as well as catechol-o-methyltransferase Val^158^Met polymorphism (*COMT*, rs4680).

Previous studies discussed the *APOE* ε4 status predominantly in the context of AD and related amyloid β (Aβ) deposits, particularly Aβ oligomers, as well as atrophies in the hippocampus [12]. Other studies linked the ε4 allele to cognition and brain structural endophenotypes, especially in late life depression [13,14,15]. It was also shown that Aβ oligomers exert a toxic influence on synaptic transmission by affecting synaptic long-term potentiation, as well as long-term depression, and might promote synapse loss in the hippocampus [15]. However, recent studies suggest that this effect might be compensated by the presence of one *BDNF*^Met^ allele [16,17], but results on this remain controversial.

*BDNF* itself is a neuronal growth factor that plays an important role in the induction of neuronal sprouting and differentiation, and is highly expressed in the hippocampus [18,19]. Especially, the single-nucleotide polymorphism *BDNF* Val^66^Met, which causes a valine (Val) to methionine (Met) substitution at codon 66 of the BDNF protein, is of high interest because of its known influence on hippocampal functions [16]. It has also been associated with AD and depression in the past [20,21]. Studies in rodents and humans suggested that carriers of the *BDNF*^Met^ allele show lower hippocampal volumes [19,22]. The differential expression of *BDNF* in different hippocampal regions might also suggest a distinct impact of the Val^66^Met polymorphism on hippocampal subfield volumes [16]. Despite these results, a joint analysis of the relation between Val^66^Met and hippocampal volumes in healthy subjects revealed no significant effect [19], and a further meta-analysis of neuropsychiatric patients showed no genotype-dependent effects [23].

Another candidate gene that might influence hippocampal volume is *KIBRA* (also known as the *WWC1* gene), which is generally connected to human memory performance [24,25,26]. As *KIBRA* interacts with synaptic proteins and is expressed in learning- and memory-related brain regions, such as the hippocampus, a link between *KIBRA* and memory performance seems plausible [25]. In addition, *KIBRA* also appears to be involved in depression-associated cognitive alterations [27]. Differences in hippocampal activation during memory retrieval were observed for *KIBRA* (rs17070145) T allele carriers, which show an increased episodic memory performance compared to non-carriers [24,28]. Other studies also observed larger hippocampal volumes for T allele carriers than for non-carriers [29,30]. Furthermore, *KIBRA* might modulate the association between hippocampal structure and episodic memory performance in combination with the gene *COMT* [31].

The *COMT* gene is located on chromosome 22 and is highly expressed in the hippocampus. Especially, the Val^158^Met polymorphism is of particular interest and plays an important role in dopamine metabolism [32] and the modulation of different brain functions [33]. The amino acid substitution of valine (Val) to methionine (Met) at codon 158 leads to a three- to fourfold reduction in enzymatic activity, which causes increased dopamine levels. Since balanced dopamine neurotransmission is necessary for the optimal regulation of human cognition, mood, and behavior, the *COMT*^Val^ allele has often been associated with poorer cognitive performance in healthy individuals [34] as well as in depressed subjects [35]. However, little is known about the impact of *COMT* on hippocampal subfield volumes. So far, studies suggest that healthy *COMT*^Val^ carriers exhibit smaller hippocampal volumes than *COMT*^Met^ carriers [36,37,38]. Hayes and colleagues [32] reported similar results for individuals with severe PTSD symptomatology.

The *5-HTTLPR* variation in the serotonin transporter gene *SLC6A4* is another prominent genetic variant, which regulates the availability of serotonin within the synaptic gap [39]. Serotonin (5-hydroxytryptamine, 5-HT) has substantial regulatory effects regarding neurite outgrowth, synaptogenesis, cell survival, and brain development. The S allele of *5-HTTLPR* is known to be associated with diminished 5-HT transporter and, consequently, 5-HT function [40]. Previous studies also reported negative effects of the *5-HTTLPR* S allele on hippocampal volumes, particularly for S allele carriers with MDD [40,41,42,43,44]. Nonetheless, again, these results are controversial [45].

However, despite plausible biological explanations for the effects of these genetic variants on hippocampal volumes, the results presented in the review by Vilor-Tejedor and colleagues [1] remain inconclusive due to the heterogeneity of samples and restricted sample sizes.

In a hypothesis-free GWAS approach, van der Meer and colleagues were able to identify 18 genome-wide significant SNPs spanning 15 loci associated either with whole hippocampal volume or specific hippocampal subfields. The genes mapped to these SNPs are involved in biological processes related to neuronal differentiation, locomotor behavior, schizophrenia, and AD [11].

The current study aimed to disentangle the effects of various genetic factors on specific hippocampal subfield volumes in a large dementia-free general population sample. The genetic factors stem from three different sources: (1) a biologically driven candidate gene approach, (2) a hypothesis-free GWAS approach, and (3) a polygenic approach, where AD risk alleles are combined with a polygenic risk score (PRS). In addition to direct effects, interaction effects between significant variants were investigated, as previous results also show moderation effects between genetic variants. In the final step, we also performed association analyses between hippocampal subfield volumes and memory performance in our sample.

## 2. Results

### 2.1. Sample Characteristic

The final sample for the calculation of direct effects between genetic variants and hippocampal subfield volumes consisted of 1806 subjects (53% females). Comparing males and females, differences in education, depression measures, verbal memory, as well as brain measures were observed (Table 1). The distribution of genetic factors was similar between both sexes (see Table 1, Supplementary Table S1). All SNPs were in Hardy-Weinberg equilibrium (HWE *p* > 0.001). A sample missingness flowchart is provided in the Supplementary Materials (see Supplementary Figure S1). A full list of GWAS SNPs can be found in the Supplementary Materials as well as a sample characteristic including all hippocampal subfields (Tables S1 and S2).

**Figure 1:**
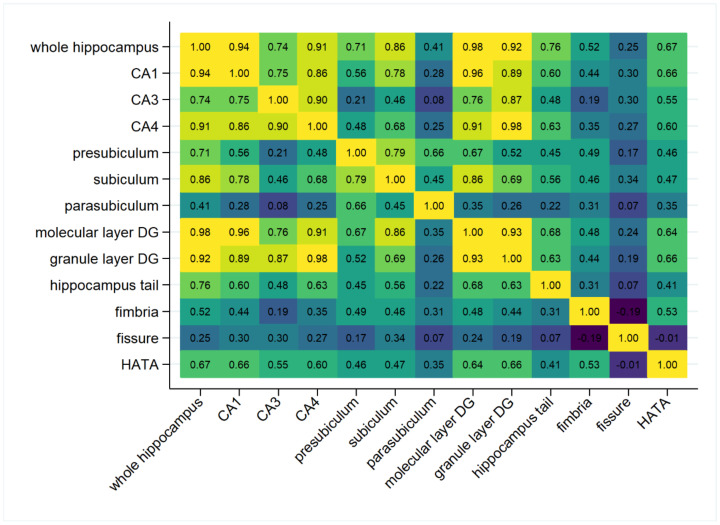
Pearson correlation heatmap of the total hippocampal volume and its subfields in TREND-0 (both hemispheres combined). CA, cornu ammonis; DG, dentate gyrus; HATA, hippocampus–amygdala transition area.

In Figure 1, the correlation matrix for total hippocampal volume and its subfields is displayed. Highest correlations with total hippocampal volume were observed for molecular layer dentate gyrus, granule layer dentate gyrus, as well as for the CA1 and CA4 subfields (all r > 0.9), the lowest correlation was for fissure (r = 0.25). The lowest absolute correlation was observed between fissure and HATA with r = −0.01. The strongest negative correlation was observed between fissure and fimbria (r = −0.19).

### 2.2. Direct Effects of Candidate Variants on Hippocampal Subfields

#### 2.2.1. *APOE* ε4

The *APOE* ε4 allele revealed negative effects on whole hippocampal volume (*p* = 0.01) as well as on different subfields (CA1, *p* = 0.003; molecular layer DG, *p* = 0.014; granule layer dentate gyrus, *p* = 0.028; HATA, *p* = 0.033; and hippocampus tail, *p* = 0.046; Table 2). As can be seen in Figure 1, this fits with the high correlations between whole hippocampal volume, CA1 region, molecular layer, and granule layer (r > 0.9).

#### 2.2.2. *5-HTTLPR*

The serotonin transporter variant revealed comparable results regarding its effects on hippocampal subfields as *APOE* ε4. The L allele was associated with a reduced whole hippocampal volume (*p* = 0.017), with negative effects on CA1 (*p* = 0.032), molecular layer DG (*p* = 0.019), granule layer DG (*p* = 0.020), and hippocampal tail (*p* = 0.009) (see Table 2).

#### 2.2.3. *BDNF*

For *BDNF* Val^66^Met polymorphism, the association pattern was largely different from those for *APOE* ε4 and *5-HTTLPR*. The Val allele was associated with reduced whole hippocampal volume (*p* = 0.038), which could especially be attributed to reductions in the presubiculum (*p* = 0.014) and subiculum (*p* = 0.025) subfield, as well as the hippocampal tail (*p* = 0.032; Table 2).

#### 2.2.4. *COMT*

No significant results were observed.

#### 2.2.5. *KIBRA*

No significant results were observed.

**Figure 2:**
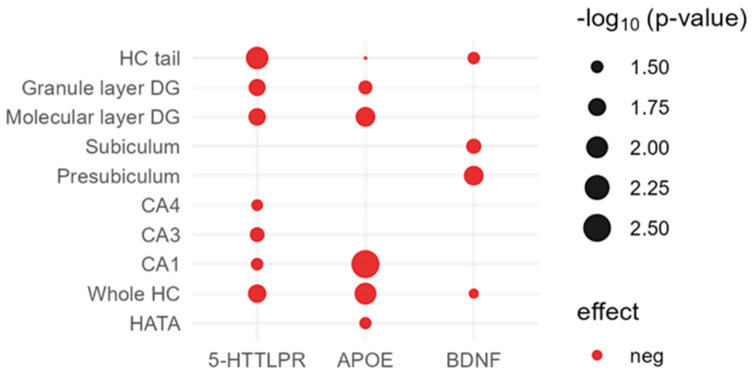
Graphical representation of all nominal significant associations between the investigated genetic candidate variants and hippocampal subfield volumes (for *COMT* and *KIBRA*, no significant association were observed). Coding alleles: *5-HTTLPR* = L allele, *APOE* = ε4 allele, *BDNF* = Val allele. CA, cornu ammonis; DG, dentate gyrus; HATA, hippocampus–amygdala transition area; HC, hippocampus.

Additional adjustment for MDD did not change the significance of the results. A graphical representation of the significant associations between candidate genetic factors and the hippocampal subfield volumes can be found in Figure 2.

### 2.3. Direct Effects of GWAS SNPs on Hippocampal Subfields

We used the 17 available genome-wide significant GWAS lead SNPs from van der Meer and colleagues [11] to look for direct effects on hippocampal subfield volumes in our sample, and to compare our results with the findings from the original publication. In TREND-0, we were able to replicate significant associations on whole hippocampal volume for four out of seven available lead SNPs from the GWAS (rs17178139, rs1861979, rs57246240, rs7873551; see Table 3 for an overview and Supplementary Table S3). From the effects on hippocampal subfields, only the effect of rs160459 on granule layer DG, of rs17178006 on CA1, and of rs2909443 on hippocampal tail could be replicated at a nominal significant level and pointing to the same effect alleles.

### 2.4. Association between the PRS for AD and Hippocampal Subfield Volumes

The PRS for AD exhibited no significant effect on any of the hippocampal subfield volumes (all *p* > 0.05).

### 2.5. Interaction Analyses between Significant Genetic Factors

Interaction effects were tested for genetic factors that exhibited a significant direct effect on hippocampal volumes. If two variants revealed significant direct effects on the same subfield, their interaction effect was tested on the same subfield. For candidate SNPs, direct effects in TREND-0 were relevant; for GWAS SNPs, we selected only those SNPs whose effect on hippocampal subfield volumes could be replicated in TREND-0. This resulted in 28 interactions that were tested (see Supplementary Table S4). None of the interactions revealed a significant effect on the respective hippocampal subfield.

### 2.6. Association between Memory Performance and Hippocampal Subfields

No significant associations between verbal memory performance and hippocampal subfields were observed in the total sample. Stratified by median age (50 years), significant associations between the delayed verbal memory score and hippocampal subfields were observed only in older subjects. Volumes of the whole hippocampus and the subfields of CA1, CA4, molecular layer DG, and granule layer DG were positively associated with a higher delayed verbal memory score. For a detailed overview, see Table 4.

## 3. Discussion

Our results reveal that genetic candidate variants selected from various biological systems show different association patterns in hippocampal subfield volumes. Hippocampal subfield volume alterations were found for *APOE* ε4, *BDNF*^Val^, and *5-HTTLPR* L allele carriers. We could also replicate part of the results from a large hippocampus subfield GWAS [11]. Interaction analyses between the significant SNPs and combining SNPs to a PRS for AD revealed no significant results. Moreover, our results confirm that hippocampal volume reductions are influenced by genetic variation, and have an impact on memory performance, especially in older age groups [46].

In order to integrate our findings into the current state of research, we searched for pathways and biological processes involving the genetic candidate variants that might influence hippocampal subfield volumes, and searched for explanations regarding how this might finally influence memory performance. As previous studies suggest, *APOE* [47], *BDNF* [48], and *5-HTTLPR* [49] might be important factors in neuroplasticity and neurodegeneration. While neuroplasticity is generally understood as the ability of neural brain networks to change through development and rearrangement [50], neurodegeneration describes the loss of neurons and synapses [47].

For *APOE* ε4 carriers, our results show reduced hippocampal volumes, especially for the dentate gyrus (granule and molecular layer) and the CA1, which play important roles in the trisynaptic circuit that represents a prominent pathway involved in information processing. It comprises different areas, such as the entorhinal cortex as well as the hippocampal subfields dentate gyrus, CA1, and CA3 [51]. It is widely assumed that sensory information initially reaches the entorhinal cortex, enters the hippocampus via the DG, and is then forwarded to CA3 and CA1 for memory consolidation [5]. To date, researchers also assume that the neuropathology of AD initially appears in the transentorhinal cortex and subsequently spreads into the entorhinal cortex and hippocampus [52]. Thus, our results are in line with the observation that *APOE* ε4 carriers are especially affected in developing AD [12]. However, how *APOE* ε4 impacts neuronal loss is still debated. Studies on AD suggest that Aβ plaques might be a common factor in the etiology of neurodegeneration in *APOE* ε4 carriers [47,53]. Here, the soluble Aβ oligomers especially appear to exert a toxic influence on synaptic transmission, and might promote neurodegenerative processes in the hippocampus [54,55]. Among others, the binding of extracellular Aβ oligomers might lead to a functional disruption of different receptor types, which results in synaptic dysfunction and neurodegeneration. Furthermore, the possible insertion of Aβ oligomers into the cell membrane and their formation of ion channels might promote neurodegenerative processes [54].

In contrast to *APOE* ε4, our observation of decreased hippocampal subfield volumes in *BDNF*^Val^ carriers initially appears counterintuitive. To date, the Met allele has been identified to decrease the activity-dependent secretion of *BDNF* due to an interference with the intracellular trafficking [56], and was associated with reduced *BDNF* levels, smaller hippocampal volumes, as well as impaired cognitive function [57]. Thus, we assumed that there were decreased hippocampal subfield volumes for Met allele carriers. Nevertheless, inconsistent findings about *BDNF* Val^66^Met are not uncommon, and might be attributable to factors such as age, sex, ethnicity, environmental factors, genetic modeling, gene–gene interactions, and non-linear genetic effects [48]. The BDNF precursor proBDNF was found to be able to induce cell apoptosis in cultured neurons [58], AD cases [59], and post-stroke depression cases [60] through the interaction with the P75^NTR^ receptor and its co-receptor sortilin; however, it still remains unclear whether proBDNF^Val^ carriers are at higher risk than proBDNF^Met^ carriers, making more research on this topic necessary. Nevertheless, our observed volume reductions for the subiculum, presubiculum, and hippocampus tail might substantiate these observations.

In addition, connections between BDNF and the serotonin transporter (5-HTTLPR) have also been discussed in the context of hippocampal neurogenesis and neurodegeneration [49]. Regions of high 5-HTTLPR concentrations are, among others, the hippocampal molecular layer and CA3 [61]. As previous studies suggest, 5-HTTLPR activity and BDNF levels are able to regulate each other through reciprocal feedback loops and enhance the growth and survival of neurons [62,63]. For instance, high levels of BDNF release result in the stimulation of 5-HTTLPR activity, which increases 5-HT clearance. As a consequence, the 5-HT receptor activity and activation of CREB (cAMP response element-binding protein) become reduced, which, in turn, leads to decreased *BDNF* expression. Vice versa, lower BDNF levels downregulate 5-HTTLPR activity and increase the expression of *BDNF* [63] via enhanced 5-HT receptor activity and activation of CREB. Since BDNF acts as a factor for neurogenesis and is associated with the growth, differentiation, and survival of neurons [64], disturbances of the homeostatic equilibrium of 5-HTTLPR and BDNF might explain hippocampal volume alterations [63]. One proposal for how this homeostasis might be impaired is provided by Haase and Brown [63]. They refer to inflammatory processes driven by infections or stressors, and describe the cytokine-induced upregulation of 5-HTTLPR activity, which leads to the depletion of BDNF levels and alterations in neuroplasticity. If so, the effect should be especially strong for *5-HTTLPR* L allele carriers. This idea is supported by the observations that the intake of selective serotonin reuptake inhibitors (SSRIs) is associated with hippocampal neurogenesis (including increased volumes, cell proliferation, and synaptic plasticity) [64], and proves to be particularly effective for L allele carriers [65,66]. By blocking 5-HTTLPR, SSRIs intervene in the mentioned homeostatic equilibrium and, thus, facilitate the expression of BDNF [63].

In our study, neither *COMT* nor *KIBRA* genetic variants revealed a significant effect on hippocampal subfield volumes. Reasons for this might be limited power due to a restricted sample size and small effect sizes of SNPs, negligible effects in the population-based sample compared to clinical cohorts, or the cross-sectional design where progressive effects are not captured. Beyond this, structural MRI data for the hippocampus can only reveal neurodegenerative effects due to differences in volume, and effects on structural plasticity and functional differences might be missed.

Concerning the GWAS lead SNPs, we found strong significant associations in our sample, especially for rs17178139 (*MSRB3*), rs1861979 (*DPP4*), rs7873551 (*ASTN2*), and rs572246240 (*MAST4*), which are in line with the recent GWAS as well as a previous meta-analysis by Hibar and colleagues [2] on hippocampus total volume. Moreover, *MSRB3* seems to be involved in processes of cell proliferation [67] and synaptic plasticity [68], *DPP4* might impact the development of oxidative stress and inflammation, which are both associated with the development of depression [69], and *ASTN2* also seems to play a role in synaptic plasticity [70] as well as in neurodevelopmental disorders [71]. Although previous knowledge about *MAST4* is scarce, *MSRB3*, *DPP4*, and *ASTN2* might be of particular interest for further candidate gene approaches.

The attempt to identify interactions between significant genetic variants as well as the application of a PRS for AD revealed no significant results.

Our findings have to be seen in the light of several limitations. Our analyses are performed on SHIP data, which are derived from a highly homogeneous general population sample of mainly European ancestry. Since we had no replication sample available, we cannot generalize our results or make any assertions as to whether our results are transferable to other cohorts and populations. Moreover, the quality of the MRI scans was limited by the resolution of the 1.5 T scanner used in this study. In addition, our conclusions are based on genetic effects and lack support from mechanistic wet lab experiments. Therefore, it is necessary to further investigate the hypothesized biological mechanisms affecting hippocampal subfields in disease samples and animal models.

In sum, our results potentially demonstrate an association between genetic variants and a reduction in certain hippocampal subfield volumes in a population-based cohort. To our knowledge, we are the first to analyze the impact of several different genetic predictors on hippocampal subfield volumes in such a structured manner using one target sample, correcting for several potential covariates, and including complex genetic variants, such as the *APOE* ε4 status or *5-HTTLPR*. We were able to identify association patterns of individual SNPs that can be linked to biological processes in neurodegeneration in a relatively healthy and dementia-free population. Whether these patterns can be transferred to disease populations needs to be clarified in independent disease cohorts. Thus, this study underlines the importance of gene-based analyses in the quest for insights into hippocampal volume loss, memory impairment, and neurodegenerative diseases, and opens the field for generating hypotheses for neurodegenerative diseases.

## 4. Materials and Methods

### 4.1. SHIP-TREND Sample

SHIP-TREND is a general population cohort from the Study of Health in Pomerania (SHIP) [72]. From 2008 until 2012, the baseline sample SHIP-TREND-0 (hereafter referred to as TREND-0) was examined with the aim of assessing the prevalence and incidence of common diseases and their risk factors in the general population. All 4420 participants underwent a standardized computer-assisted personal interview, during which they provided information on sociodemographic and lifestyle factors, and also provided different biofluids for OMICS analyses. A subsample of n = 2047 participants also underwent whole-body magnetic resonance imaging (MRI).

The investigations in the SHIP study were carried out in accordance with the Declaration of Helsinki, including written informed consent from all participants. The survey and study methods were approved by the Ethics Committee at the University Medicine Greifswald, Germany.

#### 4.1.1. Verbal Memory

In TREND-0, the word list of the Nuremberg Age Inventory (NAI) was used as a measure for immediate and delayed verbal memory performance. The NAI is a German test developed to measure cognitive abilities during brain aging [73,74]. It includes, among other subtests, a list of eight neutral words that are read to the participant, who is asked to immediately recall as many words as possible (immediate verbal memory score). After 20 min, the participant is asked to retrieve the eight words previously learned from a reading list containing eight additional distractor words. The number of correctly identified words is summarized as the sum score minus the number of wrongly identified distractor words (delayed verbal memory score).

#### 4.1.2. Covariates

In TREND-0, a diagnostic interview for mental disorders was performed based on the diagnostic criteria outlined in the Diagnostic and Statistical Manual for Mental Disorders (IV edition) [75,76], which also includes the diagnosis of lifetime major depressive disorder (MDD). Current depressive symptoms were assessed in TREND-0 using the patient health questionnaire (PHQ-9), a nine-item self-report questionnaire with high reliability and validity [77] where individual symptom load is summed up to a score ranging from 0 to 27. Education, measured as the number of schooling years, was divided into three categories according to the German school system: less than 10 years, exactly 10 years, and more than 10 years.

### 4.2. Genetic Data

#### 4.2.1. Genome-Wide SNP Chip

Genotyping of a subset of the TREND-0 subjects (n = 986) was performed using the Illumina Infinium HumanOmni 2.5 Bead Chip. The remaining TREND-0 sample (n = 3134) was genotyped at a later stage using the Illumina Infinium GSA. Imputation of genotypes was performed using the HRCv1.1 reference panel and the Eagle and minimac3 software implemented in the Michigan Imputation Server for pre-phasing and imputation, respectively. For more detail, see Völzke and colleagues [72]. SNPs with a Hardy–Weinberg equilibrium *p*-value < 0.0001, a call rate < 0.95, and a MAF < 1% were removed before imputation. 

#### 4.2.2. *APOE* ε4 Carrier Status

The *APOE* (apolipoprotein E) genotypes were determined on the basis of the two single-nucleotide polymorphisms rs429358 (C; T) and rs7412 (T; C) from the resulting imputation (imputation quality > 0.8; Hardy–Weinberg equilibrium, *p* > 0.05) [15,78]. As we used the data from the genome-wide SNP chip instead of strand-specific genotyped SNPs for the determination of *APOE* status, two ambiguous SNP combinations occurred where *APOE* ε2/ε4 and ε1/ε3 could not be discriminated (http://www.snpedia.com/index.php/APOE; accessed on 18 October 2022). Those participants were excluded from the genetic analyses (n = 99 in the total TREND-0 sample). Subjects were defined as *APOE* ε4 carriers if they had at least one ε4 allele.

#### 4.2.3. Genotyping of the Serotonin Transporter

The *SLC6A4* gene harbors a variable number tandem repeat (VNTR) polymorphism in its transcription control region (*5-HTTLPR*). Both variants (Short, Long) differ by a 43 bp insertion/deletion (“biallelic” *5-HTTLPR*). Within the inserted fragment, an additional common SNP occurs (rs25531). This finding suggested that *5-HTTLPR* is triallelic, with S, LA, and LG alleles. We developed a restriction fragment length polymorphism (RFLP) method that allows for the determination of both variants (S/L; rs25531) within one assay. For further methodological details, see the Supplementary Materials of Van der Auwera and colleagues [57].

Based on previous reports on gene expression, we also classified the genotypes into three functional ‘‘triallelic’’ genotypes: LALA = LL; LGLA or SLA = SL; and LGLG or LGS or SS = SS [58]. As the results for the “biallelic“ and “triallelic“ versions of the *5-HTTLPR* were comparable, we only report results for the triallelic version. The *5-HTTLPR* genotype was available for n = 3345 subjects in TREND-0.

#### 4.2.4. Polygenic Risk Score for AD

A polygenic risk score (PRS) is a statistical genetic measurement that sums an individual’s risk-increasing alleles weighted by their estimated effect size for a specific phenotype or disease. The PRS employed in this study was calculated using PRS-CS, a method that utilizes a high-dimensional Bayesian regression framework and places a continuous shrinkage (CS) prior on SNP effect sizes using GWAS summary statistics and an external linkage disequilibrium (LD) reference panel [79]. Here, the original effect sizes were taken from a GWAS by Kunkle and colleagues [80] on genetic risk factors for diagnosed AD. The LD reference panel was constructed using a European subsample of the UK Biobank [81]. For the remaining parameters, the default options as implemented in PRS-CS were adopted.

### 4.3. MRI Data

Subjects from TREND-0 were asked to participate in a whole-body MRI assessment. After exclusion of subjects who refused participation or fulfilled exclusion criteria for MRI (e.g., cardiac pacemaker) 2047 subjects from TREND-0 underwent the MRI scanning procedure and provided data [82]. For structural MRI data, participants were scanned with a 1.5 Tesla MRI (MAGNETOM Avanto; Siemens Healthcare, Erlangen, Germany) with a T1-weighted magnetization-prepared rapid acquisition gradient echo (MPRAGE) sequence and the following parameters: axial plane, repetition time = 1900 ms, echo time = 3.4 ms, flip angle = 15°, original resolution = 1.0 × 1.0 × 1.0 mm^3^, matrix = 256 × 176, bandwidth = 130 Hz/Pixel [83]. The brain phenotypes, including total intracranial volume, total hippocampus volume, and hippocampal subfield volumes, were generated using FreeSurfer version 7.1.1 [84]. The subfields comprised the following volumes: cornu ammonis (CA1, CA2/CA3 (referred to as CA3), CA4); presubiculum, subiculum, and parasubiculum; dentate gyrus (granule and molecular layer); hippocampal fissure, fimbria, and tail; as well as hippocampus–amygdala transition area (HATA).

### 4.4. Statistical Analyses

Subject characteristics were assessed by means, standard deviations and ranges for metric variables and with numbers and percentages for categorical data. Sample comparisons were performed using *t*-tests for metric variables and χ^2^ tests for categorical variables.

#### 4.4.1. Direct Effects of Genetic Markers on Hippocampal Subfields

Ordinary least square (OLS) linear regression models with robust estimates were applied to investigate the association of different genetic variants and the AD PRS with hippocampal subfield volumes. These genetic variants included the following candidate variants: *APOE* ε4 allele carrier status, the *BDNF* Val^66^Met polymorphism (rs6265), the *COMT* Val^158^Met polymorphism (rs4680), the triallelic version of the *5-HTTLPR*, and the *KIBRA* polymorphism rs17070145, as well as 17 lead SNPs (rs77956314 not available in our data) spanning 15 loci from a recent GWAS on hippocampal subfield volumes in the UK Biobank [11]. Analyses were adjusted for age, sex, genetic batch, three genetic principal components (PCs), education level, and intracranial volume (ICV). As many of the genetic variants were known to be associated with MDD, we also adjusted for lifetime MDD status in sensitivity analyses. The final model was the following:

*HSF ~ SNP + age + sex + batch + PC_1–3_ + education + ICV + (MDD)*

where *HSF* denotes the hippocampal subfield volumes and *SNP* the genetic variants tested. 

#### 4.4.2. Interaction Analyses between Significant Genetic Factors from Section 4.4.1

For the significant genetic factors in Section 4.4.1, we additionally performed interaction analyses with the respective hippocampal structure as the outcome to assess a possible moderation effect. GWAS SNPs were included in the models if they could be replicated in TREND-0 for the respective hippocampal subfield. The model was the same as in Section 4.4.1, except that it included two genetic components and their interaction term:

*HSF ~ SNP_1_ × SNP_2_ + age + sex + batch + PC_1–3_ + education + ICV + (MDD)*

where *HSF* denotes the hippocampal subfield volumes and *SNP* the genetic variants tested.

#### 4.4.3. Association between Memory Performance and Hippocampal Subfields

OLS linear regression analyses with robust estimates were performed to assess the association between verbal memory scores (NAI) and hippocampal subfield volumes. Analyses were performed for immediate and delayed verbal memory scores, respectively, and adjusted for age, sex, ICV, education, and current depressive symptoms, as these are highly correlated with current memory performance [15]. As age-related effects on hippocampal volume loss and associated memory impairments are common, we additionally tested the effects of advanced age by splitting the sample into young versus old subjects (median split at age > 50 years) [85]. The final model was the following:

*HSF ~ NAI + age + sex + education + ICV + PHQ-9*

where *HSF* denotes the hippocampal subfield volumes and *NAI* the scores of the Nuremberg Age Inventory.

All reported *p*-values are two-sided. In all analyses, age was treated non-linearly as cubic splines with four knots. For all genetic variants, linear effects on hippocampal subfield volumes were assumed. As this was an exploratory approach aiming to replicate or disprove previous findings, the significance level was set to *p* = 0.05. All reported analyses were performed with STATA (v. 14.2) [86].

## Figures and Tables

**Table 1:** Sample characteristics (n = 1806) for the investigated TREND-0 sample.

	Females (n = 955)	Males (n = 851)	Comparison
Age	50.6 (13.5), [21–81]	50.4 (14.3), [21–81]	T = −0.23, *p* = 0.82
Education			χ^2^ = 23.5, *p* < 0.001
<10 years	139 (14.6%)	115 (13.5%)	
=10 years	567 (59.4%)	425 (49.9%)	
>10 years	249 (26.0%)	311 (36.6%)	
Hippocampal volume in cm^3^	6.6 (0.57), [4.5–8.3]	7.2 (0.67), [5.0–9.7]	T = 20.7, *p* < 0.001
ICV in cm^3^	1496 (116), [1054–1914]	1693 (131), [1314–2135]	T = 33.8, *p* < 0.001
MDD lifetime (yes)	219 (22.9%)	106 (12.5%)	χ^2^ = 33.9, *p* < 0.001
Missing	8 (0.8%)	4 (0.5%)	
PHQ-9 sum score	4.3 (3.68), [0–25]	3.2 (3.36), [0–26]	T = −6.2, *p* < 0.001
Missing	66 (6.9%)	53 (6.2%)	
Immediate verbal memory score	5.54 (1.26), [0–8]	5.25 (1.22), [0–8]	T = −5.07, *p* < 0.001
Delayed verbal memory score	6.03 (1.59), [−3–8]	5.51 (1.68), [–1, 8]	T = −6.82, *p* < 0.001
Missing	6 (0.6%)	9 (1.1%)	
APOE ε4 status			χ^2^ = 0.48, *p* = 0.49
ε4 allele carrier	217 (22.7)	204 (24.0%)	
missing	22 (2.3%)	24 (2.8%)	
*5-HTTLPR*			χ^2^ = 1.99, *p* = 0.37
SS	202 (21.2%)	159 (18.7%)	
SL	439 (46.0%)	395 (46.4%)	
LL	224 (23.5%)	215 (25.3%)	
Missing	90 (9.4%)	82 (9.6%)	
*COMT* Val158Met			χ^2^ = 2.83, *p* = 0.24
GG (Val/Val)	254 (26.6%)	252 (29.6%)	
GA (Val/Met)	505 (52.9%)	418 (49.1%)	
AA (Met/Met)	196 (20.5%)	181 (21.3%)	
*BDNF* Val66Met			χ^2^ = 3.68, *p* = 0.16
AA (Met/Met)	36 (3.8%)	41 (4.8%)	
GA (Val/Met)	274 (28.7%)	270 (31.7%)	
GG (Val/Val)	645 (67.5%)	540 (63.5%)	
*KIBRA* rs17070145			χ^2^ = 0.60, *p* = 0.74
TT	105 (11.0%)	92 (10.8%)	
CT	416 (43.6%)	357 (42.0%)	
CC	434 (45.4%)	402 (47.2%)	

Full sample characteristics for the main analyses on the association between genetic variants and hippocampal subfields. Metric variables are displayed as mean (standard deviation) and range, categorical variables as count/number (percentage). ICV, intracranial volume; MDD, major depressive disorder; PHQ-9, Patient Health Questionnaire. The *t*-tests and χ^2^ tests were calculated on non-missing data.

**Table 2:** Association results for the direct effects of candidate genetic variants and polygenic risk score for Alzheimer’s disease (PRS AD) on hippocampal volume and its subfield volumes in TREND-0 (n = 1806).

HC Volume	*APOE*	*5-HTTLPR*	*BDNF*	*COMT*	*KIBRA*	PRS AD
**Whole HC**	**0.010(**−**)**	**0.017(**−**)**	**0.038(**−**)**	0.180 (−)	0.370 (−)	0.564 (−)
**CA1**	**0.003(**−**)**	**0.032(**−**)**	0.310 (−)	0.200 (−)	0.540 (−)	0.450 (−)
**CA3**	0.110 (−)	**0.026(**−**)**	0.640 (+)	0.440 (−)	0.680 (+)	0.346 (+)
**CA4**	0.071 (−)	**0.034(**−**)**	0.210 (−)	0.410 (−)	0.390 (−)	0.800 (+)
**Presubiculum**	0.450 (−)	0.740 (−)	**0.014(**−**)**	0.480 (−)	0.580 (−)	0.208 (−)
**Subiculum**	0.190 (−)	0.400 (−)	**0.025(**−**)**	0.500 (−)	0.230 (−)	0.347 (−)
**Parasubiculum**	0.770 (+)	0.740 (+)	0.170 (−)	0.910 (−)	0.560 (−)	0.742 (+)
**Molecular layer DG**	**0.014(**−**)**	**0.019(**−**)**	0.084 (−)	0.210 (−)	0.500 (−)	0.555 (−)
**Granule layer DG**	**0.028(**−**)**	**0.020(**−**)**	0.140 (−)	0.320 (−)	0.500 (−)	0.911 (−)
**HC tail**	**0.046(**−**)**	**0.009(**−**)**	**0.032(**−**)**	0.210 (−)	0.170 (−)	0.926 (−)
**Fimbria**	0.350 (−)	0.670 (−)	0.910 (−)	0.960 (+)	0.180 (+)	0.327 (−)
**Fissure**	0.790 (+)	0.810 (+)	0.400 (+)	0.580 (+)	0.520 (−)	0.351 (+)
**HATA**	**0.033(**−**)**	0.510 (−)	0.840 (−)	0.500 (−)	0.640 (+)	0.539 (−)

The *p*-values and effect directions (in brackets; positive + and negative −) are presented, significant results are displayed in bold. Analyses were adjusted for age, sex, intracranial volume, educational attainment, three genetic PCs, and genetic batch. Coding alleles: *5-HTTLPR* = L allele, *APOE* = ε4 allele, *BDNF* = Val allele, *COMT* = Met allele, *KIBRA* = C allele. HC, hippocampus; CA, cornu ammonis; DG, dentate gyrus; HATA, hippocampus–amygdala transition area.

**Table 3:** Comparison of significant GWAS hits with nominal significant associations in TREND-0 (n = 1806).

Lead SNP (Effect Allele)	Mapped Genes	Sig. Subfields GWAS	Sig. Subfields TREND-0
rs12218858 (C)	*FAM175B, FAM53B,* *METTL10*	Whole HC (+)	HC tail (+)
rs1419859 (C)	*PARP11*	Whole HC (+)	Subiculum (−)
rs17178139 (G)	*MSRB3*	Whole HC (+)	Whole HC, CA1, CA3, CA4, Molecular layer DG, Granule layer DG (all +)
rs160459 (A)	*DACT1*	CA1 (−), Granule layer DG (−), HC tail (+)	CA3, CA4, Granule layer DG (all −)
rs6675690 (T)	*/*	HC tail (−)	None
rs10888696 (G)	*DMRTA2, FAF1, CDKN2C*	HC tail (−)	CA1, CA3, Fissure (all −)
rs1861979 (T)	*DPP4*	Whole HC (+)	Whole HC, CA4, Granule layer DG, HC tail (all +)
rs7630893 (C)	*ATP1B3, TFDP2*	Whole HC (+)	Fimbria (−)
rs57246240 (G)	*MAST4*	Whole HC (−)	Whole HC, CA1, CA3, CA4, Presubiculum, Subiculum, Molecular layer DG, Granule layer DG, HC tail, Fissure (all −)
rs13188633 (C)	*/*	HC tail (+)	CA3, HATA (all −)
rs10474356 (A)	*/*	HC tail (+)	None
rs55736786 (C)	*FAM172A, POU5F2*	HC tail (+)	None
rs9399619 (G)	*SAMD5*	Subiculum (+)	None
rs7873551 (T)	*ASTN2*	Whole HC (+)	Whole HC, CA1, CA4, Subiculum, Molecular layer DG, Granule layer DG, HC tail, HATA (all +)
rs4962694 (G)	*FAM175B, FAM53B,* *METTL10*	Molecular layer DG (−)	HC tail (+)
rs17178006 (T)	*WIF1, LEMD3, MSRB3*	CA1 (+), Presubiculum (−)	Whole HC, CA1, Molecular layer DG, HC tail (all +)
rs2909443 (G)	*SLC4A10, DPP4*	HC tail (+)	Whole HC, CA4, Granule layer DG, HC tail (all +)

Effect directions (in brackets) are presented. Analyses were adjusted for age, sex, intracranial volume, educational attainment, three genetic principal components, and genetic batch. HC, hippocampus; CA, cornu ammonis; DG, dentate gyrus; HATA, hippocampus–amygdala transition area. rs77956314 is not available in our dataset. “/” = unknown gene.

**Table 4:** Association results between the verbal memory scores and hippocampal subfield volumes in TREND-0 (n = 1806).

Volume	Short-Term Retrieval	Long-Term Retrieval	Long-Term Retrieval (Young)	Long-Term Retrieval (Old)
**Whole HC**	0.550 (+)	0.100 (+)	0.740 (−	**0.017 (+)**
**CA1**	0.930 (+)	0.160 (+)	0.540 (−)	**0.015 (+)**
**CA3**	0.320 (+)	0.210 (+)	0.940 (+)	0.120 (+)
**CA4**	0.340 (+)	0.071 (+)	10.000 (+)	**0.027 (+)**
**Presubiculum**	0.650 (+)	0.120 (+)	0.550 (+)	0.100 (+)
**Subiculum**	0.620 (+)	0.170 (+)	0.400 (−)	**0.018 (+)**
**Parasubiculum**	0.660 (+)	0.350 (+)	0.510 (+)	0.430 (+)
**Molecular layer DG**	0.690 (+)	0.080 (+)	0.710 (+)	**0.009 (+)**
**Granule layer DG**	0.360 (+)	0.060 (+)	0.980 (−)	**0.019 (+)**
**HC tail**	0.980 (+)	0.970 (+)	0.760 (−)	0.760 (+)
**Fimbria**	0.490 (+)	0.330 (+)	0.610 (+)	0.310 (+)
**Fissure**	0.330 (−)	0.530 (+)	0.510 (−)	0.280 (+)
**HATA**	0.350 (+)	0.870 (+)	0.410 (−)	0.310 (+)

The *p*-values and effect directions (in brackets, positive +, negative −) are presented, significant results are displayed in bold. Analyses were adjusted for age, sex, intracranial volume, educational attainment, and PHQ-9 sum score. HC, hippocampus; CA, cornu ammonis; DG, dentate gyrus; HATA, hippocampus–amygdala transition area. The sample was separated by median split (median = 50 years).

## Data Availability

Research data are available after formal application to the SHIP review board (https://www.fvcm.med.uni-greifswald.de/cm_antrag/index.php, accessed on 20 May 2022).

## Supplementary Materials

The following supporting information can be downloaded at https://www.mdpi.com/article/10.3390/ijms24021120/s1.
