# Supplementary material for: Deciphering the Effect of Different Genetic Variants on Hippocampal Subfield Volumes in the General Population
Source: Int J Mol Sci. 2023 Jan 6;24(2):1120. doi: 10.3390/ijms24021120 (PMC9861136; doi:10.3390/ijms24021120)
Supplement: Supplementary file 1 [file ijms-24-01120-s001.zip › ijms-2139189-supplementary.pdf]

Supplementary Material

Deciphering the effect of different genetic variants on hippocampal subfield volumes in the general population

Kevin Kirchner, Linda Garvert, Katharina Wittfeld, Sabine Ameling, Robin Bülow, Henriette Meyer zu Schwabedissen, Matthias Nauck, Henry Völzke, Hans J. Grabe, Sandra Van der Auwera

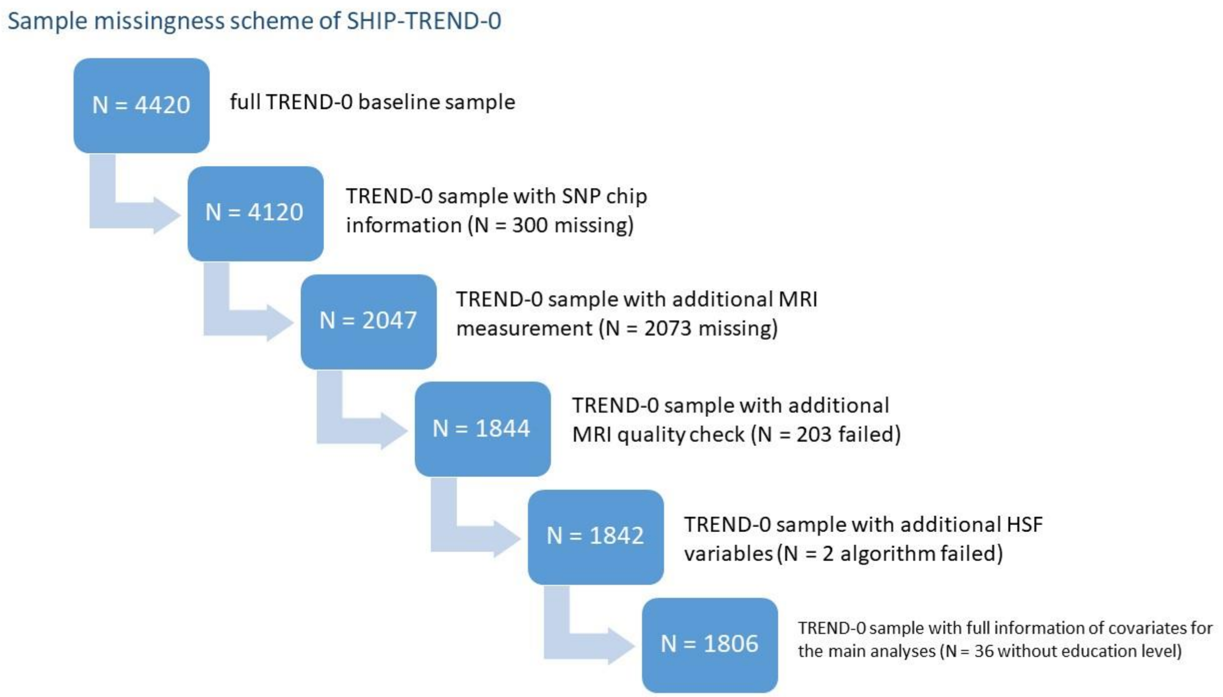

Figure S1. Sample missingness flowchart of SHIP-TREND-0

Table S1. Distribution of GWAS lead SNPs and AD PRS in TREND-0 (*n* = 1806)

	Females (n = 955)	Males (n = 851)	Comparison
**GWAS lead SNPs**			
**rs12218858**			Chi2 = 0.56, *p* = 0.76
**TT**	320 (34%)	299 (35%)	
**CT**	470 (49%)	406 (48%)	
**CC**	165 (17%)	146 (17%)	
**rs1419859**			Chi2 = 2.82, *p* = 0.25
**TT**	346 (36%)	338 (40%)	
**CT**	483 (51%)	398 (47%)	
**CC**	126 (13%)	115 (13%)	
**rs17178139**			Chi2 = 0.33, *p* = 0.85
**AA**	54 (6%)	52 (6%)	
**AG**	349 (36%)	317 (37%)	
**GG**	552 (58%)	482 (57%)	
**rs160459**			Chi2 = 0.43, *p* = 0.81
**CC**	193 (20%)	165 (19%)	
**CA**	467 (49%)	429 (50%)	
**AA**	295 (31%)	257 (30%)	
**rs6675690**			Chi2 = 0.57, *p* = 0.75
**GG**	39 (4%)	29 (3%)	
**GT**	317 (33%)	283 (33%)	
**TT**	599 (63%)	539 (64%)	
**rs10888696**			Chi2 = 4.03, *p* = 0.13
**AA**	165 (17%)	120 (14%)	
**GA**	448 (47%)	429 (50%)	
**GG**	342 (36%)	302 (36%)	
**rs1861979**			Chi2 = 0.89, *p* = 0.64
**CC**	318 (33%)	292 (34%)	
**TC**	485 (51%)	414 (49%)	
**TT**	152 (16%)	145 (17%)	
**rs7630893**			Chi2 = 2.82, *p* = 0.24
**AA**	525 (55%)	437 (52%)	
**AC**	340 (36%)	335 (39%)	
**CC**	90 (9%)	79 (9%)	
**rs57246240**			Chi2 = 3.32, *p* = 0.19
**AA**	125 (13%)	123 (14%)	
**AG**	445 (47%)	420 (49%)	
**GG**	385 (40%)	308 (36%)	
**rs13188633**			Chi2 = 0.29, *p* = 0.86
**TT**	76 (8%)	67 (8%)	
**TC**	382 (40%)	351 (41%)	
**CC**	497 (52%)	433 (51%)	
**rs10474356**			Chi2 = 7.39, *p* = 0.025
**GG**	87 (9%)	77 (9%)	
**GA**	418 (44%)	321 (38%)	
**AA**	450 (47%)	453 (53%)	
**rs55736786**			Chi2 = 2.55, *p* = 0.28
**TT**	11 (1%)	4 (0.5%)	
**TC**	140 (15%)	124 (14.5%)	
**CC**	804 (84%)	723 (85)	
**rs9399619**			Chi2 = 1.07, *p* = 0.58
**TT**	287 (30%)	275 (32%)	
**TG**	488 (51%)	421 (50%)	
**GG**	180 (19%)	155 (18%)	
**rs7873551**			Chi2 = 2.81, *p* = 0.25
**CC**	39 (4%)	46 (5%)	
**CT**	337 (35%)	277 (33%)	

**TT**	579 (61%)	528 (62%)	
**rs4962694**			Chi2 = 0.41, p = 0.81
**TT**	322 (34%)	298 (35%)	
**TG**	466 (49%)	411 (48%)	
**GG**	167 (17%)	142 (17%)	
**rs17178006**			Chi2 = 1.10, p = 0.58
**GG**	9 (1%)	6 (1%)	
**GT**	153 (16%)	150 (17%)	
**TT**	793 (83%)	695 (82%)	
**rs2909443**			Chi2 = 0.89, p = 0.64
**AA**	318 (33%)	292 (34%)	
**AG**	485 (51%)	414 (49%)	
**GG**	152 (16%)	145 (17%)	
**PRS AD**	0.266 (0.257)	0.270 (0.261)	T = 0.37, p = 0.71

rs77956314 not available in our data. PRS AD: polygenic risk score for Alzheimer's Dementia (based on Kunkle et al., 2019 [1])

Table S2. Sample characteristic for the hippocampal subfield volumes in TREND-0 (n = 1806)

	Females (n = 955)	Males (n = 851)	Comparison
**Hippocampal subfield in cm^3**			
**CA1**	1.23 (0.13), [0.8-1.6]	1.36 (0.15), [1.0-1.9]	T = 20.0, p < 0.001
**CA3**	0.41 (0.05), [0.3-0.6]	0.45 (0.06), [0.3-0.7]	T = 17.8, p < 0.001
**CA4**	0.48 (0.04), [0.3-0.6]	0.52 (0.05), [0.4-0.7]	T = 18.7, p < 0.001
**Presubiculum**	0.58 (0.07), [0.3-0.8]	0.63 (0.08), [0.4-0.9]	T = 17.4, p < 0.001
**Subiculum**	0.82 (0.08), [0.5-1.1]	0.90 (0.10), [0.6-1.3]	T = 19.9, p < 0.001
**Parasubiculum**	0.12 (0.02), [0.05-0.2]	0.13 (0.02), [0.08-0.3]	T = 12.8, p < 0.001
**Molecular layer DG**	1.07 (0.10), [0.7-1.4]	1.17 (0.12), [0.8-1.6]	T = 20.4, p < 0.001
**Granule layer DG**	0.56 (0.05), [0.4-0.7]	0.60 (0.06), [0.4-0.8]	T = 17.8, p < 0.001
**HC tail**	1.08 (0.13), [0.7-1.5]	1.14 (0.14), [0.7-1.8]	T = 8.9, p < 0.001
**Fimbria**	0.14 (0.03), [0.04-0.2]	0.16 (0.04), [0.04-0.3]	T = 9.1, p < 0.001
**Fissure**	0.28 (0.06), [0.16-0.56]	0.32 (0.06), [0.18-0.54]	T = 14.4, p < 0.001
**HATA**	0.11 (0.02), [0.06-0.16]	0.12 (0.02), [0.07-0.17]	T = 13.7, p < 0.001

All volumes are bilateral. Mean (standard deviation) and range is given. HC = hippocampus, CA = cornu ammonis, DG = dentate gyrus, HATA = hippocampus-amygdala transition area.

Table S3. Direct effects of SNPs from hippocampal subfield GWAS on hippocampal subfield volumes in TREND-0 (n = 1806).

Lead SNP	Whole HC	CA1	CA3	CA4	Presubiculum	Subiculum	Parasubiculum	Molecular layer (DG)	Granule layer (DG)	HC tail	Fimbria	Fissure	HATA
**rs12218858 (C)**	0.144 (+)	0.942 (−)	0.624 (+)	0.232 (+)	0.188 (+)	0.340 (+)	0.073 (+)	0.387 (+)	0.160 (+)	**0.015 (+)**	0.811 (+)	0.292 (−)	0.630 (+)
**rs1419859 (C)**	0.143 (−)	0.121 (−)	0.950 (−)	0.503 (−)	0.245 (−)	**0.042 (−)**	0.458 (−)	0.173 (−)	0.386 (−)	0.705 (−)	0.247 (−)	0.211 (−)	0.288 (−)
**rs17178139 (G)**	**0.003 (+)**	**0.001 (+)**	**0.003 (+)**	**0.004 (+)**	0.386 (+)	0.315 (+)	0.895 (−)	**0.003 (+)**	**0.001 (+)**	0.074 (+)	0.447 (+)	0.748 (−)	0.089 (+)
**rs160459 (A)**	0.345 (−)	0.138 (−)	**0.044 (−)**	**0.022 (−)**	0.788 (−)	0.678 (−)	0.297 (+)	0.289 (−)	**0.009 (−)**	0.111 (+)	0.304 (−)	0.803 (−)	0.068 (−)
**rs6675690 (T)**	0.674 (+)	0.409 (+)	0.748 (−)	0.816 (+)	0.277 (+)	0.202 (+)	0.285 (−)	0.393 (+)	0.733 (+)	0.353 (−)	0.701 (−)	0.792 (+)	0.793 (+)
**rs10888696 (G)**	0.101 (+)	**0.030 (+)**	**0.047 (+)**	0.144 (+)	0.545 (+)	0.107 (+)	0.739 (+)	0.081 (+)	0.125 (+)	0.944 (−)	0.955 (+)	**0.010 (+)**	0.240 (+)
**rs1861979 (T)**	**0.028 (+)**	0.208 (+)	0.397 (+)	**0.022 (+)**	0.415 (+)	0.166 (+)	0.539 (−)	0.135 (+)	**0.043 (+)**	**<0.001 (+)**	0.739 (+)	0.229 (+)	0.787 (+)
**rs7630893 (C)**	0.777 (−)	0.870 (+)	0.953 (−)	0.903 (+)	0.451 (+)	0.796 (+)	0.845 (−)	0.657 (−)	0.897 (−)	0.571 (−)	**0.027 (−)**	0.376 (+)	0.583 (−)
**rs57246240 (G)**	**<0.001 (−)**	**0.005 (−)**	**0.028 (−)**	**0.006 (−)**	**0.008 (−)**	**<0.001 (−)**	0.070 (−)	**0.001 (−)**	**0.007 (−)**	**<0.001 (−)**	0.576 (−)	**0.045 (−)**	0.151 (−)
**rs13188633 (C)**	0.200 (−)	0.395 (−)	**0.017 (−)**	0.115 (−)	0.686 (−)	0.712 (−)	0.513 (−)	0.287 (−)	0.134 (−)	0.349 (−)	0.328 (+)	0.175 (−)	**0.021 (−)**
**rs10474356 (A)**	0.260 (+)	0.549 (+)	0.508 (+)	0.267 (+)	0.371 (+)	0.232 (+)	0.866 (−)	0.401 (+)	0.385 (+)	0.290 (+)	0.624 (+)	0.806 (+)	0.847 (+)
**rs55736786 (C)**	0.976 (+)	0.777 (−)	0.950 (+)	0.792 (−)	0.284 (−)	0.807 (−)	0.112 (−)	0.717 (−)	0.946 (−)	0.186 (+)	0.550 (+)	0.199 (−)	0.672 (+)
**rs9399619 (G)**	0.289 (+)	0.908 (+)	0.277 (+)	0.195 (+)	0.489 (+)	0.160 (+)	0.765 (+)	0.404 (+)	0.362 (+)	0.334 (+)	0.918 (−)	0.517 (+)	0.564 (+)
**rs7873551 (T)**	**0.002 (+)**	**0.036 (+)**	0.071 (+)	**0.015 (+)**	0.235 (+)	**0.007 (+)**	0.976 (−)	**0.011 (+)**	**0.010 (+)**	**0.009 (+)**	0.050 (+)	0.686 (−)	**0.011 (+)**
**rs4962694 (G)**	0.207 (+)	0.757 (−)	0.766 (+)	0.322 (+)	0.202 (+)	0.418 (+)	0.085 (+)	0.521 (+)	0.231 (+)	**0.018 (+)**	0.772 (+)	0.300 (−)	0.676 (+)
**rs17178006 (T)**	**0.025 (+)**	**0.002 (+)**	0.169 (+)	0.196 (+)	0.932 (−)	0.320 (+)	0.832 (−)	**0.023 (+)**	0.066 (+)	**0.042 (+)**	0.735 (+)	0.506 (+)	0.346 (+)
**rs2909443 (G)**	**0.028 (+)**	0.208 (+)	0.397 (+)	**0.022 (+)**	0.415 (+)	0.166 (+)	0.539 (−)	0.135 (+)	**0.043 (+)**	**<0.001 (+)**	0.793 (+)	0.229 (+)	0.787 (+)

p-values and effect directions (in brackets) are given, nominal significant results in TREND-0 are displayed in bold. Highlighted in yellow: genome-wide significant associations from the GWAS van der Meer et al. (2020). For significant results effect directions were identical to van der Meer et al. (2020). Analyses adjusted for age, sex, intracranial volume, educational attainment, three genetic principal components and genetic batch. HC = hippocampus, CA = cornu ammonis, DG = dentate gyrus, HATA = hippocampus-amygdala transition area. rs77956314 not available in our dataset. Effect alleles for each SNP are given in brackets.

Table S4. Interaction effects of candidate SNPs × GWAS SNPs [2] on hippocampal subfield volumes in TREND-0 (n = 1806).

Interaction	Whole HC	CA1	Molecular layer (DG)	Granule layer (DG)	HC tail
**APOE × BDNF**	0.258 (−)	/	/	/	0.147 (−)
**APOE × 5-HTTLPR**	0.498 (−)	0.347 (−)	0.688 (−)	0.761 (−)	0.603 (−)
**APOE × rs160459**	/	/	/	0.966 (−)	/
**APOE × rs2909443**	/	/	/	/	0.679 (−)
**APOE × rs1861979**	0.798 (+)	/	/	/	/
**APOE × rs7873551**	0.364 (+)	/	/	/	/
**APOE × rs57246240**	0.083 (−)	/	/	/	/
**APOE × rs17178139**	0.260 (+)	0.415 (+)	/	/	/
**5-HTTLPR × BDNF**	0.605 (−)	/	/	/	0.607 (−)
**5-HTTLPR × rs160459**	/	/	/	0.730 (+)	/
**5-HTTLPR × rs2909443**	/	/	/	/	0.191 (−)
**5-HTTLPR × rs1861979**	0.142 (−)	/	/	/	/
**5-HTTLPR × rs7873551**	0.134 (+)	/	/	/	/
**5-HTTLPR × rs57246240**	0.093 (−)	/	/	/	/
**5-HTTLPR × rs17178139**	0.205 (+)	0.407 (+)	/	/	/
**BDNF × rs2909443**	/	/	/	/	0.783 (−)
**BDNF × rs1861979**	0.220 (+)	/	/	/	/
**BDNF × rs7873551**	0.498 (+)	/	/	/	/
**BDNF × rs57246240**	0.146 (−)	/	/	/	/
**BDNF × rs17178139**	0.881 (−)	/	/	/	/

p-values and effect directions (in brackets; positive + and negative −) are given, significant results are displayed in bold. Analyses adjusted for age, sex, intracranial volume, educational attainment, three genetic principal components and genetic batch. HC = hippocampus, CA = cornu ammonis, DG = dentate gyrus. APOE = *APOE* ε4 status; BDNF = Val^{66}MET polymorphism.

References

1. Kunkle, B.W.; Grenier-Boley, B.; Sims, R.; Bis, J.C.; Damotte, V.; Naj, A.C.; Boland, A.; Vronskaya, M.; van der Lee, S.J.; Amlie-Wolf, A.; et al. Genetic meta-analysis of diagnosed Alzheimer's disease identifies new risk loci and implicates Aβ, tau, immunity and lipid processing. *Nat. Genet.* **2019**, *51*, 414–430, doi:10.1038/s41588-019-0358-2.
2. van der Meer, D.; Rokicki, J.; Kaufmann, T.; Córdova-Palomera, A.; Moberget, T.; Alnæs, D.; Bettella, F.; Frei, O.; Doan, N.T.; Sønderby, I.E.; et al. Brain scans from 21,297 individuals reveal the genetic architecture of hippocampal subfield volumes. *Mol. Psychiatry* **2020**, *25*, 3053–3065, doi:10.1038/s41380-018-0262-7.
